# A synthetic genetic array screen for interactions with the RNA helicase *DED1* during cell stress in budding yeast

**DOI:** 10.1093/g3journal/jkac296

**Published:** 2022-11-21

**Authors:** Sara B Carey, Hannah M List, Ashwin Siby, Paolo Guerra, Timothy A Bolger

**Affiliations:** Department of Molecular and Cellular Biology, University of Arizona, Tucson, AZ 85721, USA; Department of Molecular and Cellular Biology, University of Arizona, Tucson, AZ 85721, USA; Department of Molecular and Cellular Biology, University of Arizona, Tucson, AZ 85721, USA; Department of Molecular and Cellular Biology, University of Arizona, Tucson, AZ 85721, USA; Department of Molecular and Cellular Biology, University of Arizona, Tucson, AZ 85721, USA

**Keywords:** yeast, helicase, translation, stress, rapamycin

## Abstract

During cellular stress it is essential for cells to alter their gene expression to adapt and survive. Gene expression is regulated at multiple levels, but translation regulation is both a method for rapid changes to the proteome and, as one of the most energy-intensive cellular processes, a way to efficiently redirect cellular resources during stress conditions. Despite this ideal positioning, many of the specifics of how translation is regulated, positively or negatively, during various types of cellular stress remain poorly understood. To further assess this regulation, we examined the essential translation factor Ded1, an RNA helicase that has been previously shown to play important roles in the translational response to cellular stress. In particular, *ded1* mutants display an increased resistance to growth inhibition and translation repression induced by the TOR pathway inhibitor, rapamycin, suggesting that normal stress responses are partially defective in these mutants. To gain further insight into Ded1 translational regulation during stress, synthetic genetic array analysis was conducted in the presence of rapamycin with a *ded1* mutant and a library of nonessential genes in *Saccharomyces cerevisiae* to identify positive and negative genetic interactions in an unbiased manner. Here, we report the results of this screen and subsequent network mapping and Gene Ontology-term analysis. Hundreds of candidate interactions were identified, which fell into expected categories, such as ribosomal proteins and amino acid biosynthesis, as well as unexpected ones, including membrane trafficking, sporulation, and protein glycosylation. Therefore, these results provide several specific directions for further comprehensive studies.

## Introduction

During adverse extracellular conditions, such as nutrient deprivation or oxidative stress, cells must reorient their gene expression profiles to slow growth, conserve resources, and respond to the stressor (Pakos-Zebrucka *et al.* 2016; Saxton and Sabatini 2017). Translation is both highly energy-intensive and a direct determinant of the cellular proteome; thus, it is a natural point of regulation in stress responses (Liu and Qian 2014; Crawford and Pavitt 2019). Indeed, translation undergoes massive reprogramming during stress, wherein bulk translation is repressed, but translation of select “stress–response” mRNAs is upregulated (Ingolia *et al.* 2009; Gerashchenko *et al.* 2012). However, the mechanisms underlying this specificity remain incompletely understood.

In budding yeast, *DED1* encodes an essential RNA helicase of the DEAD-box protein family, which are critical for modulating RNA–RNA and RNA–protein interactions throughout gene expression (Valentini and Linder 2021). Its human ortholog, *DDX3X*, has been implicated in multiple cancers, including frequent mutations in medulloblastoma, and *DDX3X* mutations also cause an autism-like cognitive disorder (Northcott *et al.* 2012; Snijders Blok *et al.* 2015; Mo *et al.* 2021; Tang *et al.* 2021). The primary function of Ded1 is thought to be in translation initiation. In normal, progrowth conditions, Ded1 promotes initiation by unwinding secondary structure in the 5′ UTR of mRNAs and stimulating preinitiation complex assembly (Sharma and Jankowsky 2014; Sen *et al.* 2015; Gupta *et al.* 2018). Furthermore, *ded1* mutation preferentially affects mRNAs with structured 5′ UTRs and increases the utilization of alternative translation initiation sites in target mRNAs (Sen *et al.* 2015; Guenther *et al.* 2018).

Interestingly, Ded1 also plays roles in repressing translation. *DED1* overexpression inhibits translation and cell growth, and Ded1 affects the formation of stress granules, stress-dependent, cytoplasmic accumulations of RNA and proteins (Beckham *et al.* 2008; Hilliker *et al.* 2011; Aryanpur *et al.* 2022). Notably, we recently showed that Ded1 mediates the translational response to TOR inactivation (Aryanpur *et al.* 2019). Specifically, a *ded1* mutant lacking its C-terminal region (*ded1-ΔCT*) was resistant to growth inhibition and translation repression caused by the TOR inhibitor rapamycin. The C-terminal region of Ded1 interacts with the scaffolding factor eIF4G1, and further analysis suggested that eIF4G1 mediates the effects of Ded1 in these conditions (Hilliker *et al.* 2011; Aryanpur *et al.* 2019). We proposed a model wherein Ded1 represses translation during TOR inactivation by promoting eIF4G1 dissociation from translation complexes and its subsequent degradation. How this mechanism is regulated and which downstream processes are affected remains unknown, however.

To begin to address these questions, we conducted a synthetic genetic array (SGA) screen with the *ded1-ΔCT* mutant, taking advantage of its resistance to rapamycin-mediated growth inhibition to identify both positive and negative synthetic interactions from the yeast deletion library of nonessential genes (Tong *et al.* 2001). This screen identified a large number of synthetic interactions with associated Gene Ontology (GO) terms that include translation, vesicle trafficking, amino acid metabolism, and signal transduction. These hits likely represent upstream regulators, direct interactions, and downstream targets of Ded1 as well as related processes. Compelling candidates will be examined in future studies.

## Materials and methods

### Screen design

The yeast strain used in this SGA screen is TBY174 (*MAT*α *his3*Δ*1 leu2*Δ*0 ura3*Δ*0 can1*Δ*0∷P_GAL1_-T_ADH1_-P_MFA1_-spHIS5 lyp1*Δ*0 ded1-ΔCT::Hygro*), which was constructed from the strain Y15583-13.2b from a previous study using similar techniques (Singh *et al.* 2009). The C-terminal portion of *DED1* was removed from Y15583-13.2b and replaced with a hygromycin resistance cassette using the plasmid pUG75 as a template [protocol adapted from Hegemann and Heick (2011)]. The resulting strain was verified using PCR amplification, growth assays, and western blotting. This “query strain” (TBY174) was then used in a standard SGA protocol (Tong and Boone 2006; Singh *et al.* 2009). The yeast knockout library is commercially available and contains approximately 5,000 nonessential gene knockout strains (Horizon Discovery). These strains were constructed using a G418 resistance cassette (Giaever *et al.* 2002). The mating type of the strains used are *MATa* allowing them to be mated to the query strain directly.

The screen procedure can be summarized as: rearray of the knockout library, mating of query strain to the library, selection of zygotes, sporulation, selection of haploids, and growth analysis. To rearray the library, the 96-well plates of the library stock were pinned using a RoToR HDA Pinning robot (Singer) to a 384-well format compatible with the robot. These plates contained solid YPD (10 g/l yeast extract, 20 g/l peptone, 20 g/l agar, 200 µl/l 10 N NaOH, 2% glucose) + G418 (200 mg/l) media to maintain selection, and yeast were grown for 48 h at 30°C. To prepare for mating, the query strain was simultaneously grown in liquid culture for 24 h, plated to a 384-well format, and allowed to grow for an additional 24 h at 30°C. The query strain was then replica plated to 14 fresh plates containing YPD. Next, the rearrayed knockout library plates were replica plated directly on top of the query strain and allowed to grow for 24 h at 30°C. To select for zygotes, the mated strains were replica plated to new plates containing YPD + G418 + hygromycin (200 mg/l) and allowed to grow for 48 h at 30°C. For efficient sporulation, the zygote plates were replica plated to plates containing sporulation media (20 g/l agar, 10 g/l potassium acetate, 1 g/l yeast extract, 0.5 g/l glucose, and 0.1 g/l amino acid sporulation supplement; supplement consisted of 2 g histidine, 10 g leucine, 2 g lysine, and 2 g uracil) and allowed to grow for 5 days at 22°C.

Following the sporulation period, the desired double knockout (DKO) haploid strains were isolated using a progression of 3 different selections. First, *MATa* sporulated strains were selected by replica plating onto Singer-compatible plates containing SD media (20 g/l agar, 20 g/l glucose, 1.7 g/l yeast nitrogen base without ammonium sulfate and amino acids, 1 g/l monosodium glutamic acid, and 2 g/l amino acid supplement; supplement consisted of 1.2 g adenine, 1.8 g isoleucine, 3.6 g leucine, 1.2 g methionine, 3.0 g phenylalanine, 1.2 g tryptophan, 1.8 g tyrosine, 1.2 g uracil, 9 g valine, 1.5 g aspartic acid, 1.5 g glutamic acid, 1.5 g threonine, 1.5 g serine, and 1.5 g proline) lacking histidine, arginine, and lysine + canavanine (50 mg/l) + thialysine (50 mg/l) and grown for 48 h at 30°C (*MATa* cells are selected via *spHIS5* expression by the *MATa*-specific *MFA1* promoter while canavanine and thialysine select against unsporulated diploids). After 48 h these plates were replica plated onto fresh plates containing the same media as before and grown for 24 h at 30°C in order to generate “tighter” spots for subsequent steps. Second, for selection of the knockout library gene deletion, the *MATa* strains were replica plated onto plates containing SD—His/Arg/Lys + canavanine + thialysine + G418 and grown for 48 h at 30°C. Third, for selection of the *ded1-ΔCT* mutation, strains were replica plated onto plates containing SD—His/Arg/Lys + canavanine + thialysine + G418 + hygromycin and grown for 48 h at 30°C. Growth on the final set of plates generated the DKO strains used for phenotypic screening.

### Phenotype scoring

**Fig. 1.**
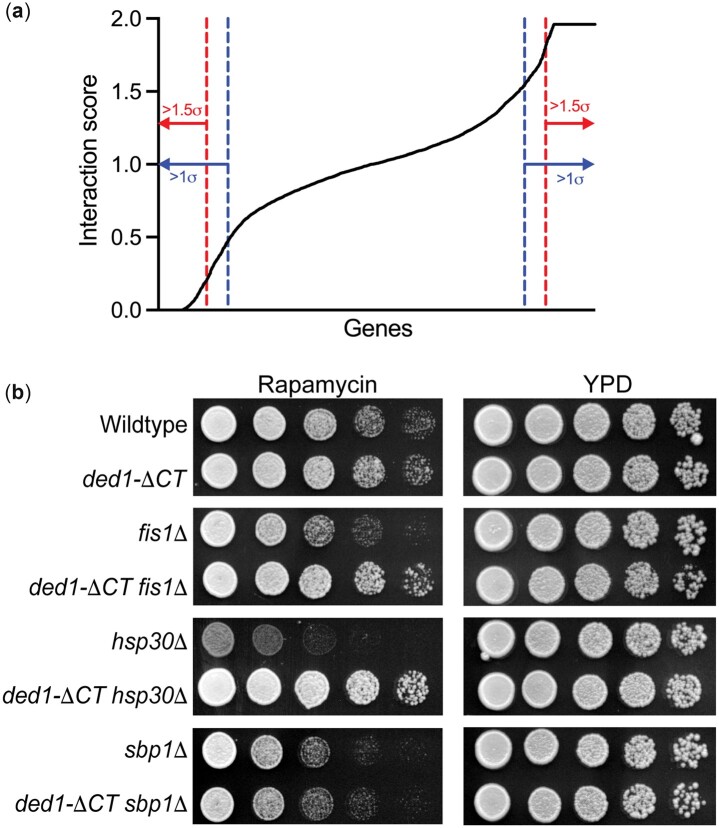
Identification of genetic interactions with *ded1-ΔCT* following rapamycin treatment. a) The synthetic interaction scores with *ded1-ΔCT* for growth on rapamycin for all genes tested (4,799) are shown in ascending order. The inner dashed blue lines represent 1 SD from the mean score, and the outer dashed red lines represent 1.5 SD. Genes falling outside of these thresholds were considered “hits” and used for further analysis. b) Growth of single (*fis1Δ*, *hsp30Δ*, and *sbp1Δ*) and *ded1-ΔCT* double mutants for 3 representative hits on rich media (YPD) and rich media plus rapamycin are shown. Five-fold serial dilutions were grown at 30°C for 2 (YPD) or 4 (Rapamycin) days. Synthetic interactions were observed consistent with the screen results.

After generation of the DKO strains, the strains were tested for growth fitness on media containing rapamycin. Both the single-mutant *ded1-ΔCT* query strain and some library deletion strains are resistant or sensitive to rapamycin [see Fig. 1b; Supplementary Table 4, and Aryanpur *et al.* (2019)]. Therefore, DKO strains were scored by normalizing growth to that of the single-mutant parent strains via a multiplicative method (Baryshnikova *et al.* 2010). Fitness was assessed for each of the following on YPD and YPD + rapamycin (200 ng/ml) media: Y15583-13.2b (as a wild-type control), *ded1-ΔCT* query strain, single knockout library strains, and DKO strains. While still plated in a 384-well format, YPD plates were grown for 2 days and YPD + rapamycin plates were grown for 5 days at 30°C. Plates were scanned using a flat-bed scanner (Epson) and colony size was analyzed via SGA Tools (http://sgatools.ccbr.utoronto.ca; Last accessed 11/9/22), taking into account all 4 strains/controls in both conditions (Wagih *et al.* 2013). SGA tools calculated fitness as the number of pixels contained in each spot for each strain. Then, using the single knockout in both conditions, the query strain in both conditions, and the wild-type control in both conditions as parameters for comparison, a normalized score was generated that represented the “interaction score” for the DKO strain on a scale from 0 to 2. This score thus provides a quantitative measure of the synthetic interaction between the *ded-ΔCT* allele and the library deletion for rapamycin-dependent growth, where 0 represents no growth of the double mutant, which is an extremely negative synthetic interaction (synthetic-lethal), 1 represents the expected growth given no interaction, and 2 represents much better growth on rapamycin than expected for the double mutant, a highly positive synthetic interaction (for technical reasons, the actual maximum was 1.961 rather than 2.000).

### Classification and verification of hits

To determine cutoffs for further analysis of the hits, the interaction scores for all 4,799 DKO strains were analyzed via Graphpad Prism. The score distribution had a mean of 1.007 and an SD of 0.534. Cutoffs were established at 1.0 and 1.5 SD above and below the mean wherein interaction scores below the lower cutoff (0.473 or 0.205, respectively) were analyzed as negative/suppressor interaction hits and scores above the upper cutoff (1.542 or 1.809) were analyzed as positive/enhancer hits. The 1.5 SD cutoff, which yielded 529 suppressor and 544 enhancer hits, was used for most subsequent analysis; however, the 1.0 SD cutoff, which yielded 763 suppressor and 780 enhancer hits, was used for generating lists of *Saccharomyces* Genome Database (SGD)-annotated phenotypes.

To experimentally verify the phenotypes, single and double mutants from a selected number of hits were isolated from the screen strains and individually tested via serial dilution growth assays on YPD and YPD + rapamycin plates at 30°C as previously described (Aryanpur *et al.* 2017). In addition, the list of hits includes a number of genes that were expected based on previous studies (e.g. translation factors).

### Network maps and GO-term analysis

Network maps were generated with the hits (1.5 SD cutoff) using STRING (https://string-db.org/; Last accessed 11/9/22) (Snel *et al.* 2000; Szklarczyk *et al.* 2021). STRING was then used to perform k-means clustering on the hits, where nodes (genes) were clustered into a predetermined number of clusters such that each node is related more strongly to other nodes within the same cluster than to nodes within other clusters. The gap statistic method was used to determine that 5 clusters should be used for each of the 2 datasets (suppressors and enhancers) (Tibshirani *et al.* 2001). We then used STRING to associate GO terms that are significantly enriched within each of the clusters. GO terms were ranked for each cluster based on the “strength” feature in the STRING analysis, where strength is defined as log_10_ of the ratio between the number of proteins in the hits that are annotated with a specific GO-term and the number of proteins that would be expected to be annotated with this term in a random network of the same size. GO-term lists were manually curated by eliminating redundant or vague terms (e.g. “cytoplasm”), and up to 10 GO terms are shown. Complete lists of all significantly enriched GO terms are included in the Supplementary Tables.

For the GO-term analysis of annotated hits (Table 3; Supplementary Tables 4 and 5), the hits (1.0 SD cutoff) were examined for a previously annotated phenotype of rapamycin (sirolimus) resistance or sensitivity using the “YeastMine” feature of SGD. Four categories of hits were thus generated: suppressors (negative synthetic interactions with *ded1-ΔCT*) with a previously annotated rapamycin sensitivity (203 hits), suppressors with annotated rapamycin resistance (54), enhancers with rapamycin sensitivity (78), and enhancers with rapamycin resistance (60). Note that only about one-quarter of the hits were annotated for a rapamycin phenotype in SGD; thus, 3 of the 4 categories were too small for effective network mapping. Instead, overrepresented GO terms (biological process complete) were generated for each category using PANTHER (pantherdb.org; Last accessed 11/9/22) via Fisher’s exact test (Mi *et al.* 2021). GO terms with less than 4 associated genes were deleted and then were curated as above with the 8 most-enriched terms shown in Table 3 (complete list in Supplementary Table 5).

**Table 1.** Enriched GO terms in cluster analysis of suppressors.

	GO term	No. of proteins	Strength	False discovery rate
Cluster 1	Tricarboxylic acid metabolic process	3	1.5	0.0357
	Re-entry into mitotic cell cycle after pheromone arrest	3	1.45	0.0439
	Branched-chain amino acid biosynthetic process	4	1.32	0.0133
	Alpha-amino acid metabolic process	21	0.98	1.26 × 10^−11^
	Dicarboxylic acid metabolic process	5	0.97	0.0399
	Mitochondrial translation	8	0.77	0.0142
Cluster 2	Phosphatidylinositol-3-phosphate biosynthetic process	4	1.55	0.0018
	Intralumenal vesicle formation	4	1.41	0.0041
	ATP export	8	1.32	2.05 × 10^−5^
	Protein retention in Golgi apparatus	4	1.21	0.0135
	Vacuolar acidification	8	1.12	0.00011
	Retrograde transport, endosome to Golgi	8	0.98	0.00044
	Phosphatidylinositol metabolic process	10	0.87	0.00025
	Vesicle organization	15	0.83	2.05 × 10^−5^
	Late endosome to vacuole transport	9	0.83	0.0012
	Vacuole organization	13	0.82	5.59 × 10^−5^
Cluster 3	Heme transport	3	1.7	0.0108
	De novo cotranslational protein folding	3	1.55	0.0214
	Structural constituent of ribosome	19	0.86	3.00 × 10^−8^
	Translation	29	0.81	1.19 × 10^−12^
	Ribosome biogenesis	17	0.55	0.0009
	RNA binding	26	0.51	2.06 × 10^−5^
	ncRNA processing	15	0.51	0.0081
Cluster 4	Regulation of conjugation with cellular fusion	8	0.82	0.0465
	Carbohydrate metabolic process	19	0.74	1.23 × 10^−5^
Cluster 5	Sucrose catabolic process	3	1.35	0.0186
	Peptidyl-histidine modification	3	1.3	0.0238
	Histone H3 acetylation	3	1.3	0.0238
	Nucleosome disassembly	6	1.26	0.0001
	Telomere tethering at nuclear periphery	5	1.22	0.001
	Histone deacetylation	7	1.19	5.00 × 10^−5^
	Posttranscriptional tethering of RNA polymerase II gene DNA at nuclear periphery	4	1.13	0.012
	Double-strand break repair via nonhomologous end joining	6	1.11	0.0006
	DNA-templated transcription, elongation	12	1.06	2.12 × 10^−7^
	Regulation of transcription by RNA polymerase I	6	0.96	0.0031

**Table 2.** Enriched GO terms in cluster analysis of enhancers.

	GO term	No. of proteins	Strength	False discovery rate
Cluster 1	Ribosome	16	0.51	0.0358
Cluster 2	Hydrolase activity, hydrolyzing O-glycosyl compounds	8	1.01	0.0036
Cluster 3	Regulation of transcription from RNA polymerase II promoter in response to oxidative stress	3	1.39	0.0449
	Peptidyl-tyrosine dephosphorylation	4	1.17	0.0293
	Regulation of MAPK cascade	5	1.04	0.0216
	Negative regulation of signal transduction	6	1	0.0119
	Regulation of signal transduction	12	0.83	0.00032
	Protein glycosylation	7	0.83	0.0194
	Cellular response to abiotic stimulus	6	0.8	0.0449
	Establishment or maintenance of cell polarity	9	0.76	0.0096
Cluster 4	Aerobic respiration	11	0.81	0.0083
Cluster 5	AP-type membrane coat adaptor complex	5	1.39	0.0045
	Late endosome	5	0.87	0.0499
	Vesicle	12	0.53	0.0366
	Bounding membrane of organelle	20	0.38	0.0366

**Table 3.** Enriched GO terms in previously annotated hits.

	GO term	No. of proteins	Strength	*P*-value
Known rapamycin-resistant enhancers	Positive regulation of GTPase activity	4	1.08	0.0005
	Negative regulation of transcription by RNA polymerase II	6	0.64	0.0026
	Response to oxidative stress	5	0.63	0.0066
	Regulation of intracellular signal transduction	4	0.62	0.0167
	Ribosomal large subunit biogenesis	4	0.51	0.0360
	Telomere organization	4	0.51	0.0360
	Chromatin organization	9	0.50	0.0022
	Positive regulation of transcription by RNA polymerase II	9	0.50	0.0022
Known rapamycin-sensitive enhancers	Positive regulation of DNA-templated transcription elongation	4	0.85	0.0029
	Dephosphorylation	4	0.77	0.0054
	Endocytosis	6	0.67	0.0021
	Protein localization to membrane	5	0.52	0.0188
	Regulation of translation	6	0.49	0.0137
	Autophagy	5	0.42	0.0419
	DNA repair	8	0.41	0.0136
	Vesicle-mediated transport	11	0.38	0.0059
Known rapamycin-resistant suppressors	Negative regulation of transcription by RNA polymerase II	9	0.90	2.03 × 10^−6^
	RNA catabolic process	5	0.64	0.0058
	Protein ubiquitination	4	0.62	0.0168
	Positive regulation of transcription by RNA polymerase II	8	0.53	0.0024
	Cellular ion homeostasis	4	0.49	0.0423
Known rapamycin-sensitive suppressors	Intralumenal vesicle formation	4	1.25	0.0003
	ATP export	9	1.22	4.63 × 10^−8^
	Protein localization to Golgi apparatus	8	1.07	1.91 × 10^−6^
	Maintenance of DNA trinucleotide repeats	4	1.05	0.0010
	Positive regulation of TOR signaling	4	0.98	0.0016
	Homoserine metabolic process	4	0.92	0.0025
	Retrograde transport, endosome to Golgi	11	0.89	7.47 × 10^−7^
	Vacuolar acidification	7	0.89	8.31 × 10^−5^

## Results and discussion

We crossed a *ded1-ΔCT* mutant to a knockout library of nonessential genes and assessed the resulting 4,799 double mutants for their growth on rapamycin-containing media. We then assigned each pair a synthetic interaction score (from 0 to 2, with 1 representing no interaction) after normalizing for growth on rapamycin of both single mutant parent strains, where a low score indicates that the double mutant grew less well on rapamycin than expected based on the single mutant phenotypes (a negative synthetic interaction), and a high score indicates that the double mutant grew better than expected (a positive synthetic interaction). The interaction scores were distributed across the range of possible scores with an overall mean of 1.007 (Fig. 1a). A large number of mutants showed strong synthetic interactions with 266 scoring at the lowest value possible and 453 at the highest, respectively. Cutoffs for candidate hits were established as any mutants with an interaction score more than 1.0 or 1.5 SD from the mean, depending on the downstream analysis (Fig. 1a; Supplementary Table 1).

Results were verified by individually testing growth of selected hits. In Fig. 1b, 3 examples are shown. The *fis1-null ded1-ΔCT* and *hsp30-null ded1-ΔCT* double mutants both grew significantly better on rapamycin than the *fis1-null* and *hsp30-null* mutants alone (positive interactions), while the *sbp1-null ded1-ΔCT* double mutant grew similarly to the *sbp1-null* mutant alone (and more poorly than the *ded1-ΔCT* single mutant), indicating a negative interaction. Thus, the growth assays agreed with the results from the screen, although we found that some strongly negative interactions were also synthetic lethal or synthetic sick in the absence of rapamycin (data not shown). Further supporting the validity of the screen, genes expected to interact were also obtained, including yeast *FKBP1* (*FPR1*) and numerous genes involved in translation (*GCN2*, ribosomal genes, etc.). It should be noted, however, that a small but significant number of the strains in the deletion collection have been shown to have off-target mutations or other defects (Giaever and Nislow 2014); therefore individual hits should be interpreted with caution.

**Fig. 2.**
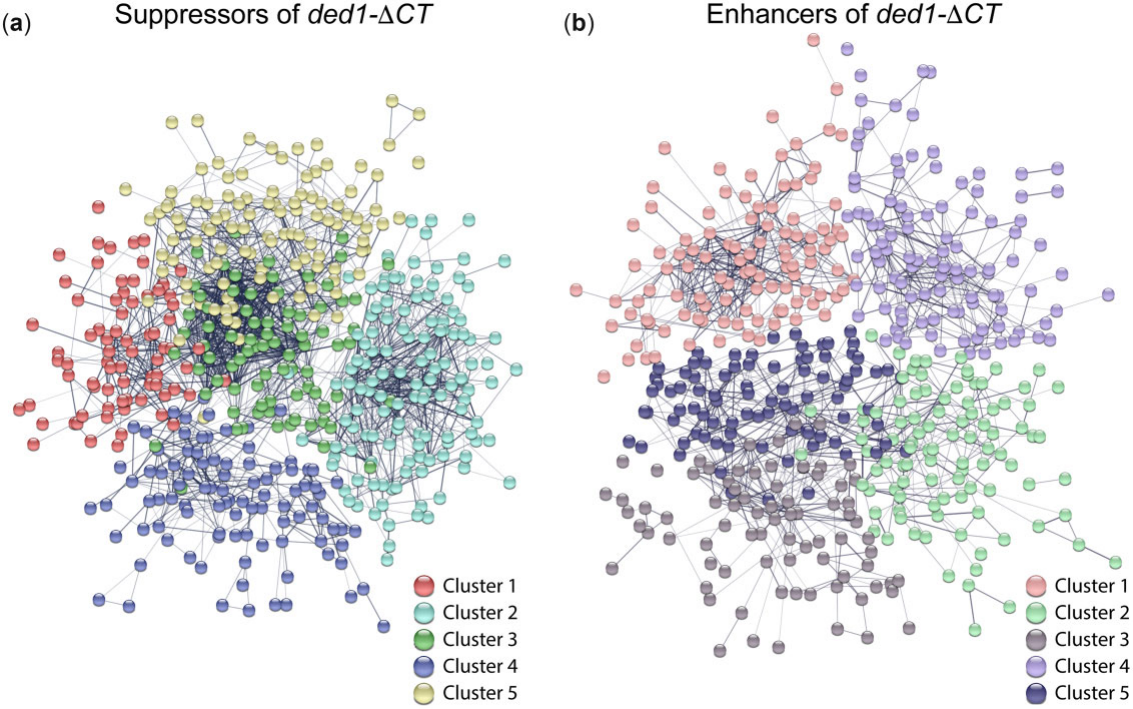
Network cluster maps of interacting genes. Network maps were generated using STRING for the synthetic negative/suppressors of *ded1-ΔCT* (a) and the synthetic positive/enhancers of *ded1-ΔCT* (b) hits that exceeded the 1.5 SD threshold below and above the mean interaction score, respectively. Thickness of the edges between genes signifies the strength of data support for the interaction. Disconnected nodes/genes are not shown. Clusters were generated via k-means clustering. The identity of each cluster (no. 1–5) is labeled below, and corresponding colors were used in Tables 1 and 2.

To organize the large number of hits from the screen, we conducted protein network analysis using STRING (Snel *et al.* 2000; Szklarczyk *et al.* 2021). An interaction network was built with all the genes showing strongly negative synthetic interactions with *ded1-ΔCT* (“suppressors”) in the presence of rapamycin (529 genes with interaction score more than 1.5 SD from the mean), and this network was then partitioned into 5 groups by *k*-means clustering (Fig. 2a). A similar network was built and clustered for the 544 genes showing strongly positive synthetic interactions (“enhancers,” Fig. 2b). These clusters were then analyzed for GO terms that are enriched in these subsets in order to determine which cellular processes and pathways interact with *DED1* most strongly during stress conditions.

### Negative interactors/suppressors of ded1-ΔCT

Tables 1 and 2 show the most enriched GO terms in each cluster (up to 10), following curation to remove highly similar terms (for complete lists of GO terms, see Supplementary Tables 2 and 3). Suppressor cluster 1 included a substantial number of genes involved in amino acid metabolism, including multiple genes involved in synthesis of several different amino acids (arginine, isoleucine, serine, etc.) as well as synthesis of complex carboxylic acids (Table 1). Amino acid synthesis pathways are often upregulated in nutrient-poor conditions, so these genes may represent downstream targets regulated by Ded1, although amino acid availability also regulates TOR activity upstream (Saxton and Sabatini 2017; Crawford and Pavitt 2019). Cluster 1 also included 8 genes involved in mitochondrial translation, which may affect energy production for translation. Suppressor cluster 2 yielded a number of GO terms that are related to membrane-mediated trafficking, including phosphatidylinositol signaling, intralumenal vesicle formation, endosomal transport, and vacuole regulation. These genes could be regulating Ded1 activity through its degradation along with its binding partner eIF4G1 during cell stress (Kelly and Bedwell 2015; Aryanpur *et al.* 2019), or they could be cross-talk from TOR-dependent regulation of autophagy and endosomal trafficking (Strahl and Thorner 2007; Saxton and Sabatini 2017; Hatakeyama *et al.* 2019). Cluster 2 also included 8 genes involved in ATP export, which may again reflect an effect on energy production.

Suppressor cluster 3 was largely focused on translation (Table 1), including 19 ribosomal proteins and at least 10 additional translation factors. Other aspects of translation were also represented, including ribosome biogenesis and noncoding RNA processing, which mostly consisted of tRNA processing genes (Supplementary Table 2). These are likely affecting the ability of Ded1 to regulate translation. Three protein chaperones that act cotranslationally as well as 3 heme transport genes were also present in this cluster. Suppressor cluster 4 yielded relatively few GO terms, including the regulation of conjugation (mating) as well as carbohydrate metabolism. We have observed that *ded1-ΔCT* mutants have somewhat delayed sporulation compared to wild-type cells (data not shown), so these interactions are consistent with a Ded1 function in yeast mating/sporulation. Suppressor cluster 5 included a more diverse set of terms, including sucrose catabolism, peptidyl-histidine modification, double-strand break repair, and several terms related to chromatin remodeling and transcription. Alterations in chromatin state and/or transcription are of course part of stress responses and could represent upstream regulation or downstream targets of Ded1 activity following TOR inactivation (Saxton and Sabatini 2017; Crawford and Pavitt 2019). The 3 peptidyl-histidine modification genes all target translation factors for modification (ribosomal proteins and elongation factors), which may explain their genetic interaction with *DED1* (Uthman *et al.* 2013; Al-Hadid *et al.* 2016).

### Positive interactors/enhancers of ded1-ΔCT

Despite a similar number of hits, the enhancer clusters yielded fewer GO terms than the suppressors, suggesting a more diverse set of genes overall (Table 2; Supplementary Table 3). Enhancer cluster 1 included a number of ribosomal and ribosomal-related proteins, showing that alterations in different proteins involved in translation have the potential to either synergize or antagonize Ded1 function during cell stress. Enhancer cluster 2 only yielded one significant GO term, for hydrolase activity. These hydrolases are all involved in cell wall regulation during both sporulation and cytokinesis following mitosis. Their link to Ded1 is unclear but may be through sporulation and/or changes to the cell cycle during stress.

Enhancer cluster 3 gave the largest number of GO terms for the enhancers overall with particular enrichment for signal transduction genes, particularly the MAPK pathway. Notably, several of these (e.g. *PTC2*, *SDP1*, *PTP2*) are phosphatases that negatively regulate the MAPK pathway (Martin *et al.* 2005); therefore, their deletion would tend to increase growth and might synergize with increased growth in the *ded1-ΔCT* mutant in rapamycin. Other GO terms in this cluster included transcriptional responses to oxidative stress, which fits well with Ded1 function in stress, and protein glycosylation, which has unclear links to Ded1. Only one GO term, aerobic respiration, was associated with enhancer cluster 4. This may again be due to energy requirements during stress. Finally, enhancer cluster 5 included several GO terms associated with membrane trafficking, similar to suppressor cluster 2, although with a more specific focus on vesicle trafficking, specifically. The relationship to Ded1 function is unclear, although these interactions may be due to TOR-dependent changes in membrane trafficking that affect Ded1 activity during stress.

### Annotation of hits by rapamycin-dependent phenotype

In theory, the positive synthetic interactions/enhancers in this screen could be generated either by synergistic effects of a rapamycin-resistant mutation and the rapamycin-resistant *ded1-ΔCT* allele (a resistant enhancer phenotype), or by suppression of a rapamycin-sensitive mutation by *ded1-ΔCT* (a sensitive enhancer phenotype). Likewise, negative synthetic interactions/suppressors could be due to suppression of *ded1-ΔCT* rapamycin resistance by a rapamycin-sensitive mutation (sensitive suppressor), or by suppression by a rapamycin-resistant mutation (resistant suppressor). To attempt to assign hits to these various categories, we mined the phenotypes of the *Saccharomyces* Genome Database for those genes with mutations annotated as resistant or sensitive to rapamycin, and then we correlated these with the genes in our screen with an interaction score more than 1.0 SD from the mean. Only a minority of the hits were annotated for a rapamycin-dependent phenotype (138 enhancers and 257 suppressors), so we were not able to conduct in-depth network analyses for these subsets. Nonetheless, we generated enriched GO terms for each of the 4 subsets, which are summarized in Table 3 (for complete lists, see Supplementary Tables 4 and 5). The resistant enhancers subset included GO terms for regulation of GTPase activity, regulation of transcription, ribosomal biogenesis, and response to stress. The sensitive enhancers subset included transcription elongation, phosphatase activity, membrane trafficking, translation, and DNA repair. The sensitive suppressors subset included the largest number of annotated hits (203) and yielded the largest number of GO terms, including intralumenal vesicle formation, other membrane trafficking terms, ATP export, TOR signaling, and DNA maintenance. By contrast, the resistant suppressors subset was the smallest with 54 genes, and GO terms included transcription regulation, ubiquitination, and ion homeostasis. Overall, the GO terms in this analysis largely corresponded to the terms from the cluster analysis above, with several new terms such as GTPase activity and ubiquitination. However, this division into subcategories may be useful in designing follow-up experiments to directly examine these interactions with *DED1*.

In this screen, we obtained a large number of potential interactions with the *ded1-ΔCT* mutant. Many of these fell into categories that would be predicted by the known functions of Ded1 during cellular stress, including ribosomal proteins and translation factors, amino acid biosynthesis genes, and transcription and chromatin remodeling factors. Some of these, such as ribosomal proteins, translation factors, and amino acid regulators, likely function together with Ded1 and/or in parallel to effect immediate translational reprogramming during stress. Likewise, mutations affecting other gene expression processes (e.g. transcription and chromatin remodeling) can presumably also indirectly affect translation through mRNA transcript abundance. Interestingly, genes encoding ribosomal proteins were identified as both enhancers and suppressors of *ded1-ΔCT*, perhaps reflecting the complexity of ribosome composition and function.

Several more categories of hits have more tangential links to the stress function of Ded1, including genes involved in membrane trafficking, signal transduction, mitochondrial/energy production genes, and sporulation genes. Stress regulation, and TOR signaling in particular, are strongly linked to changes in autophagy and other membrane trafficking processes (Saxton and Sabatini 2017), so it is not entirely surprising that genetic interactions between Ded1 and membrane trafficking components were observed. Likewise, Ded1 has been suggested previously to have a role in the regulation of sporulation (Guenther *et al.* 2018), making hits in sporulation genes also highly plausible. Notably, the identified hits in the MAPK cascade are largely negative regulators of that pathway (Supplementary Table 3), so null mutants of these factors might be expected to enhance stress-resistant growth. Follow-up experiments to explore the links between Ded1 and these processes could lead to better understanding of the coordination and regulation of cellular stress responses.

Lastly, some hits were in unexpected categories, such as cell wall hydrolases, protein glycosylation, and GTPase activity. As with other hits, hits in this category could represent upstream regulators of Ded1 (GTPases), crosstalk between cellular processes (cell wall regulation or protein glycosylation), or possibly mRNAs that are translationally targeted by Ded1 (any). Future work may be able to elucidate these interactions with *DED1* and how they contribute to stress or other cellular responses.

## Supplementary Material

jkac296_Supplementary_Data

## Data Availability

Strains and plasmids are available upon request. The Supplementary tables contain complete lists of all data and analysis from the screen, including interaction scores for all genes (Supplementary Table 1), SGD-annotated phenotypes (Supplementary Table 4), and complete lists of GO terms (Supplementary Tables 2, 3, and 5). Supplemental material is available at G3 online.
